# In vitro assessment of needle and irrigant penetration when using different irrigation needle tips

**DOI:** 10.2340/biid.v12.42896

**Published:** 2025-02-18

**Authors:** Saleha Hussain, Lars Bjørndal, Merete Markvart

**Affiliations:** Department of Odontology, University of Copenhagen, Copenhagen, Denmark

**Keywords:** Needles, syringes, root canal irrigation

## Abstract

**Objective:**

The aim of this study was to compare the needle and irrigant penetration depth of a newly developed multi-vented polymer needle (30G), with three established needle designs – an open-ended metal needle (30G), a side-vented polymer needle (30G), and a notched metal needle (27G) used as a reference control. The effect of manual dynamic activation (MDA) was also measured. The null hypotheses were that the irrigant penetration would be the same regardless of needle tip, and the addition of MDA would make no difference in terms of irrigant penetration.

**Materials and methods:**

A total of 120 mesial roots from mandibular molars were instrumented to a size 25/07, with reciprocating files. The maximum needle penetration depth was measured for each needle tip, using a rubber stop. Syringe irrigation was performed using a sodium diatrizoate solution, first with needle placement halfway down the root canal (working length subtracted from the canal length), and then 1 mm from the working length. MDA was performed. A digital radiograph was taken before the initial irrigation, after the initial irrigation, after the final irrigation, and after MDA. Digital subtraction was performed, and irrigant penetration was measured by a blinded operator. Non-parametric statistical tests were conducted using Mann–Whitney *U*-test and Wilcoxon signed-rank test.

**Results:**

The multi-vented polymer needle had a significantly deeper needle penetration (mean value: 99%), compared to other test needles. The deepest irrigant penetration was achieved using the multi-vented polymer needle (mean value: 98%) and the open-ended metal needle (mean value: 99%). A significantly deeper irrigant penetration, was achieved by adding MDA, regardless of needle tip.

**Conclusions:**

The multi-vented polymer needle and the open-ended metal needle showed superior performance in terms of irrigant penetration. However, the irrigant penetration only managed to reach the working length when MDA was added.

## Introduction

The purpose of a root canal treatment is to eliminate or prevent infection, through cleaning, shaping, and disinfection of the root canal system. The success of the procedure depends largely on the effective removal of infected tissue and thorough disinfection of the canals. Achieving these goals requires a combination of chemical disinfection through irrigants and mechanical preparation with endodontic files and instruments. Together, these two approaches form the foundation of chemo-mechanical preparation [1, 2].

Chemo-mechanical preparation involves shaping the canal to a specific diameter and taper, to ensure that irrigants can reach, and thereby cleanse and disinfect, as much of the root canal wall as possible [2]. This can be particularly challenging in cases with complex anatomical structures, such as lateral canals, and severe curvatures. Historically, larger canal preparations were common, as 27G needles were used standardly for irrigation. However, increasing the preparation size to facilitate irrigant delivery can elevate the risk of tooth fracture, cause over-instrumentation and compromise the apical seal after obturation [3]. While proper disinfection is essential for the success of any root canal treatment, preserving tooth structure is equally important for the long-term functionality, and thereby the overall prognosis of the tooth [4]. Consequently, the use of finer needle tips capable of reaching the apical third of the canal, despite a narrow preparation, has become increasingly popular [5, 6]. Although a universally accepted ‘golden standard’ needle has yet to be established, a wide variety of 30G needles are now commonly used in clinical practice, offering a balance between effective irrigation and the preservation of tooth integrity.

The final apical diameter is typically determined by the natural size and shape of the root canal. The severity of the infection, and the instrument selection can also be decisive factors [7].

Several studies have highlighted the critical role of irrigant penetration in the success of root canal treatments [8], showing that factors such as needle design, final apical diameter, and procedural techniques significantly influence the outcome [3, 7, 9]. Open-ended needles have been shown to have a deeper irrigant penetration than other designs, as the lack of vents or notches facilitates a higher pressure directed toward the apex, enabling the irrigant to reach an extensive 2–3 mm beyond the needle tip, although not without increasing the risk of apical extrusion. Side-vented needles, with a vent or set of vents along the side of the needle, direct the flow laterally, thereby reducing the risk of extrusion, however, consequently compromising the irrigant penetration as the flow is diverted away from the apical areas. For this reason, studies have recommended placing open-ended needles 2–3 mm from the working length and side-vented needles only 1 mm from the working length [10, 11]. Notched needles, are open-ended needles, designed with a cut-out on the side, and have been shown to have a slightly more localized flow, compared to the regular flat open-ended needles [3].

Despite these advancements, limited research has focused on needle tips that irrigate on multiple levels along the entire root canal simultaneously.

Regardless of the needle design, studies have consistently shown that dynamic activation of the irrigant, such as through manual dynamic activation (MDA), can improve the effectiveness of the irrigation, even in cases where the final apical diameter is kept small or in anatomically complex canals, by generating a turbulent flow [12].

**Figure 1:**
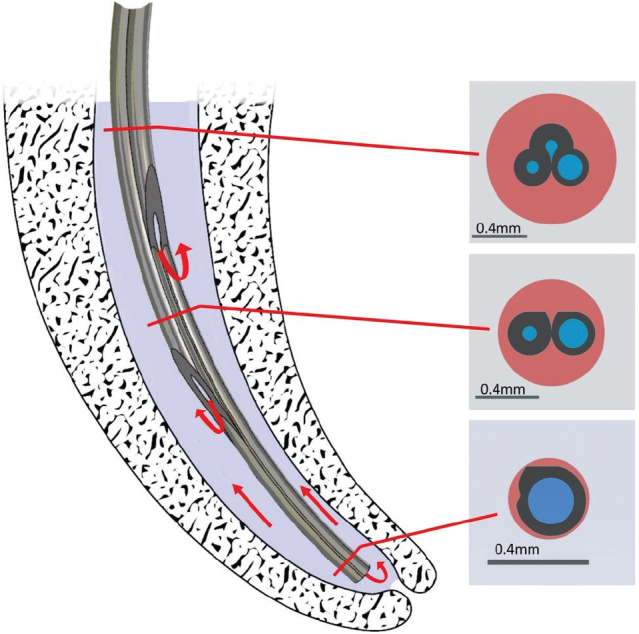
An illustration of the prototype combined with cross-section illustrations at each level corresponding to the three exit vents. The polymer needle has been designed with several intentions in mind. Three internal lumens intended to promote liquid flow from all three openings, and with different diameter to control the amount of flow. The apical lumen has the largest diameter, favoring flow toward the apical third of the canal, whereas the coronal lumens contribute to the turbulence within the return flow, with the purpose of increasing the shear stress along the root canal wall for better cleaning. The noncylindrical cross section of the needle contributes to an efficient and safe backflow since the sides of the needle is less likely to block the flow within the root. Furthermore, if debris blocks the backflow from the lower part of the needle and pressure builds up at the apex, the liquid will instead be able to flow through the coronal lumens, thus preventing an overpressure at the apex.

**Figure 2:**
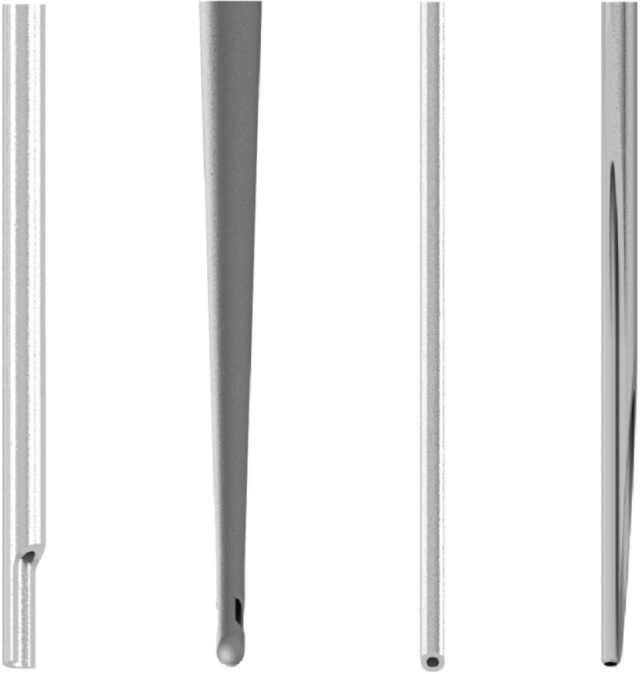
A description of the needle tips used in the experiments and an illustration presenting the needles, in following order from left to right: the notched metal needle (27G), the side-vented polymer needle (30G), the open-ended metal needle (30G), and the multi-vented polymer needle (30G). The illustration was created in Autodesk Inventor 2022 (Autodesk, Inc., San Francisco, CA, USA) and Adobe Illustrator CC (Adobe Inc., San Jose, CA, USA). The prototype, PolyNeedle, is referred to as ‘the multi-vented polymer needle’ here and in the article.

The present study compared a newly developed needle prototype, a 30-gauge polymer needle with three exit vents (Figure 1), with an open-ended metal needle and a side-vented polymer needle in terms of both needle and irrigant penetration, before and after MDA. A notched metal needle (27G) was used as a control reference for comparison (Figure 2).

The null hypotheses are: (1) that a statistically significant deeper irrigant penetration cannot be achieved by using a specific needle tip and (2) the effectiveness of the irrigation procedure will not be further enhanced by combining with MDA.

## Materials and methods

### Power calculation

Sample size calculations (MedCalc Software Ltd, Belgium) determined that 29 samples were required per group to detect a difference of ~30% in needle penetration depth, with a two-sided alpha level of 5% (type I error) and 80% power (Type II error of 20%), when expecting an approximate 2/3 penetration of the established needle designs providing an estimated large variation (S.D. 5 mm). To account for potential dropouts, one additional sample was included per group, resulting in 30 samples per group and a total of 120 teeth in the study.

### Collection and preparation of extracted teeth

A total of 120 human first and second mandibular molars with mature apices were selected for the study. The mesial canals of these teeth were chosen for testing, as they typically present a challenge, in terms of needle and irrigant penetration due to complex anatomical variations [13]. Ethics approval was not required as all patient data were anonymized. The teeth were stored at 4°C in a 0.1% thymol solution to prevent bacterial growth and dehydration.

Carious lesions were non-selectively excavated, and if needed, the residual crown was reconstructed using resin-modified glass ionomer (GC Fuji TRIAGE^®^; GC Corporation, Tokyo, Japan). Soft tissue debris and calculus was removed, and access cavities were prepared under an operating microscope (Carl Zeiss Surgical GmbH, Oberkochen, Germany). The distal roots were removed, to provide a clear proximal view of the mesial root canal configurations.

Size 10 K-files (Dentsply Maillefer, Ballaigues, Switzerland) were placed in both mesial root canals and radiographs were taken to confirm canal separation in the coronal two-thirds. Canal curvature was measured using the Schneider method [14]. Only teeth with canal lengths between 10 and 15 mm were included. Tooth length was standardized at 19 mm from the apical foramen, by decoronation using a diamond disk. Working length (WL) was determined 1 mm short of the apical foramen, at 18 mm.

The root canal instrumentation followed a four-phase standard protocol [15]. The root canals were initially instrumented with size 10 and size 15 K-files (Dentsply Maillefer, Ballaigues, Switzerland), both reaching the WL. Apical preparations were then performed using a nickel-titanium reciprocating file (WaveOne Gold; Dentsply Maillefer, Ballaigues, Switzerland). Canal calibration was performed using a fitted gutta-percha cone, to ensure all canals had an apical preparation size of #25 (0.07 taper). Irrigation was performed between instruments using 1 mL of 2.5% sodium hypochlorite, delivered through a notched 27-gauge needle (Monoject^TM^; Covidien, Tullamore, Ireland). The final irrigation was performed using 17% EDTA-C (Nordenta, Hørning, Denmark) for 1 min, followed by a rinse with 3 mL of 2.5% sodium hypochlorite, using an open-ended 30-gauge needle (Navitip^TM^; Ultradent Products, Cologne, Germany). The root canals were dried with paper points (WaveOne Gold; Dentsply Maillefer, Ballaigues, Switzerland).

Size 25 gutta-percha cones were inserted into the canals, before the roots were sealed with transparent nail varnish apically and embedded in cold-mounting resin (EpoFix; Struers, Ballerup, Denmark), to create a closed-ended system [16] The samples were stored in water at 4°C, until the day of the experiment, where the canals were dried with paper points immediately before testing.

Each tooth was assigned a curvature, canal length and a connection status (Table 1). Preoperative radiographs from a proximal and facial aspect were taken for this purpose. The teeth were randomly assigned into four groups (*n* = 30), with an equal number of teeth from each subgroup. The randomization was performed by an independent operator, using a concealed allocation sequence.

**Table 1:** Classifications of the different variables.

Variable			
*Curvature*	Moderate (*n* = 18) 10°–20° 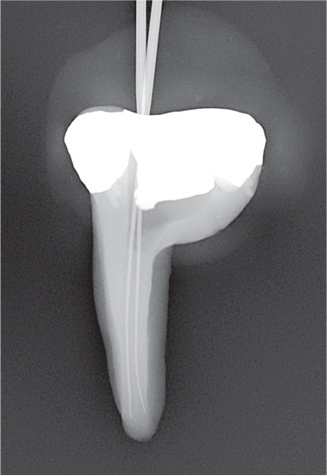	Moderate-severe (*n* = 22) 21°–24° 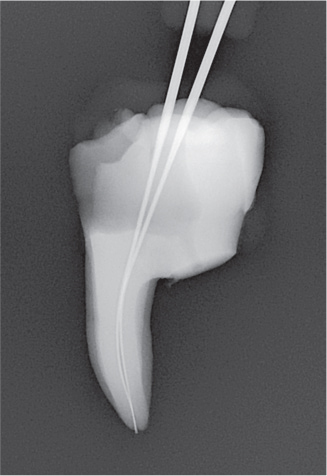	Severe (*n* = 80) 25°–70° 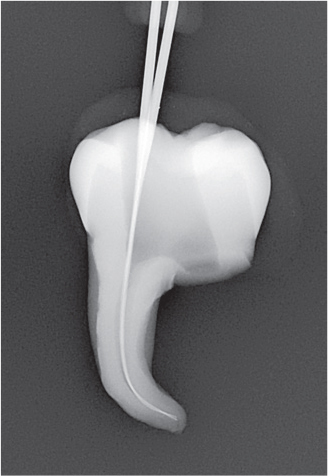
*Connection status*	Connected (*n* = 70) 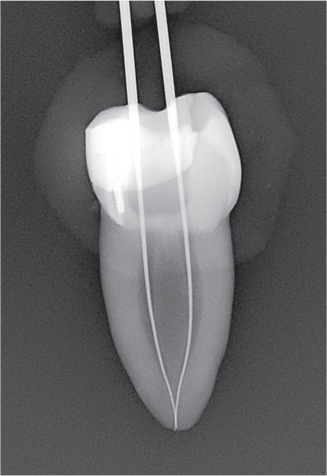	Separate (*n* = 50) 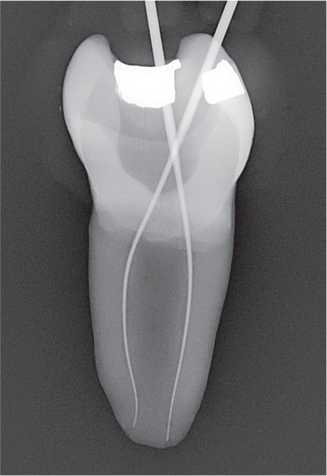	
*Canal length*	Short (*n* = 93) 10–12 mm 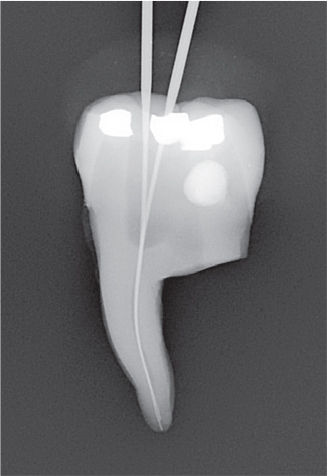	Long (*n* = 27) 12.5–15 mm 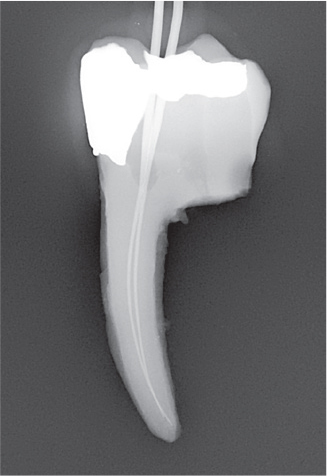	

### The irrigation experiments

Four different needle tips were selected for this study, one prototype, and three established needle tips, with one acting as a control reference (Table 2, Figure 2). Each needle tip was assigned to one of the four groups.

**Table 2:** Mean values (expressed in fractions) for each sub-experiment along with standard deviations.

Needle tips	I	II	III	IIII
Monoject^TM^; Covidien, Tullamore, Ireland ‘The notched metal needle’	0.80 (0.05)	0.83 (0.14)	0.92 (0.07)	0.98 (0.03)
TruNatomy^TM^; Dentsply Maillefer, Ballaigues, Switzerland ‘The side-vented polymer needle’	0.97 (0.03)	0.84 (0.10)	0.97 (0.02)	0.99 (0.01)
Navitip^TM^; Ultradent Products, Cologne, Germany ‘The open-ended metal needle’	0.92 (0.05)	0.88 (0.09)	0.99 (0.03)	1.00 (0.01)
PolyNeedle; DTU, Kongens Lyngby, Denmark ‘The multi-vented polymer needle’	0.99 (0.02)	0.87 (0.12)	0.98 (0.02)	1.00 (0.01)

The teeth embedded in resin, a radiographic reference ball (2.5 mm in diameter) and a CCD sensor (Plandent, Helsinki, Finland) were attached to a custom-made mould screwed into a rectangular X-ray tube, to obtain images with identical projection geometry, as needed for digital subtraction radiography. Radiographs were taken from the mesiodistal aspect, to assess irrigant penetration and distribution.

**Figure 3:**
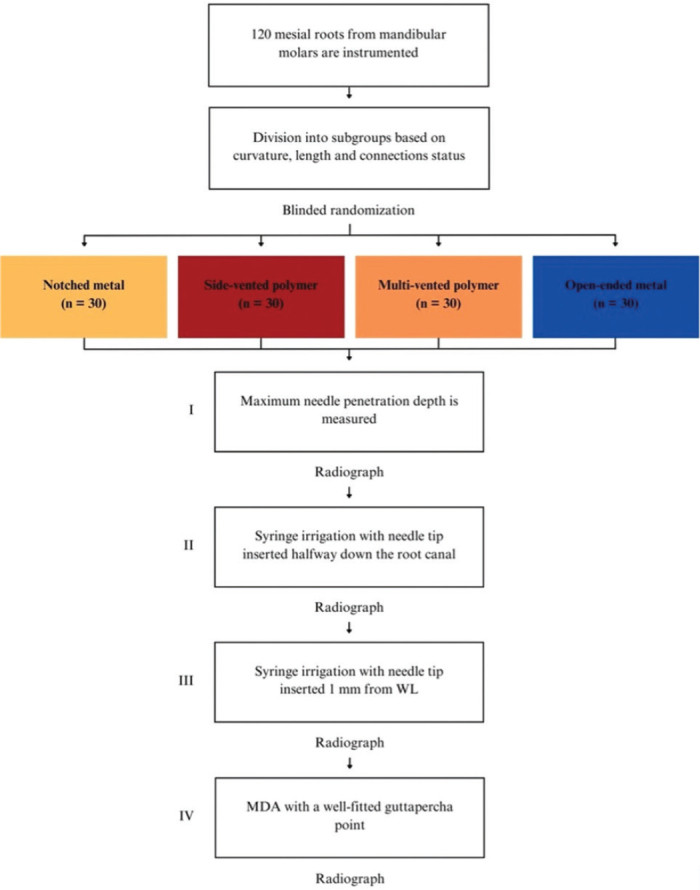
Flowchart of the irrigation experiment.

The experiment was conducted in four parts (Figure 3).

- The needle tip was placed passively to the maximum attainable insertion depth and the needle penetration depth was measured using a rubber stop.
- The needle tip was placed halfway down the root canal (WL subtracted from 1/2 of the canal length) and syringe irrigation was performed using 0.5 mL of sodium diatrizoate (Hypaque sodium 50%; Acros Organics, Geel, Belgium) per canal, in a 3 mL syringe.
- The needle tip was placed 1 mm from the WL without binding, or if not possible, at the maximum attainable insertion depth, and syringe irrigation was again performed using 0.5 mL of sodium diatrizoate.
- A fitted gutta-percha was inserted at WL and moved perpendicularly in three cycles of 20 s each, using vertical strokes with an amplitude of 2–3 mm, at a rate of ~ 100 strokes/min [12].

A digital radiograph was taken between each part and each part was repeated once per tooth using one needle tip per tooth. The order of testing was randomized to minimize any procedural bias.

### Digital subtraction

**Figure 4:**
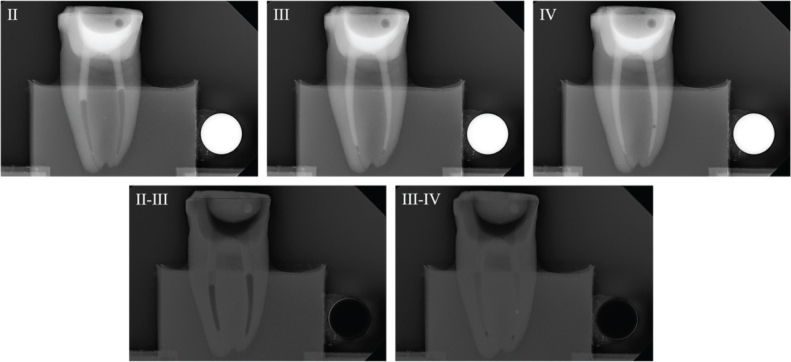
Representative radiographs from the irrigation experiments and the corresponding digital subtraction images. Images II, III and IV represent the study parts of the irrigation experiments, respectively. Image II-III illustrates image from part II subtracted from image from part III, and image III-IV illustrates image from part III subtracted from image from part IV. For analysis of the part II, image II was used, where the irrigant was measured from an occlusal reference point. For analysis of part III, image II-III was used where the difference in irrigant penetration was measured and added to the previous measurement. For analysis of part IV, image II-IV was used, where the difference in irrigant penetration was measured and added to the first and second measurement. The subtraction images were inversed, for optimal visualization.

Radiographs were imported into Adobe Photoshop 28 (Adobe Inc., San Jose, CA, USA) for digital subtraction. A blinded operator assessed the irrigant penetration by comparing the radiographs from each part of the four parts (I–IV) (Figure 4). The results were presented as a fraction of the WL.

### Statistical analysis

As the data were not normally distributed (determined through histograms), non-parametric statistical tests were conducted. Mann–Whitney *U* tests were conducted to compare needle and irrigant penetration depth across the needle tips for the first, second and third part of the experiments. The null hypothesis for this comparison was that the needle and irrigant penetration would be the same regardless of needle tip.

To assess the effect of MDA, irrigant penetration with needle placement 1 mm from WL was compared to irrigant penetration after MDA, within the same needle tip, using Wilcoxon signed-rank test. The null hypothesis for this analysis was that MDA would not significantly increase the irrigant penetration. Significance levels for all statistical analysis were set at 0.05. Statistical analysis was performed using SPSS Statistics 28 (IBM Corporation, Armonk, NY, USA).

## Results

Mean values and standard deviations are presented for all experiments (Table 2).

### I. Maximum attainable needle penetration depth

**Figure 5:**
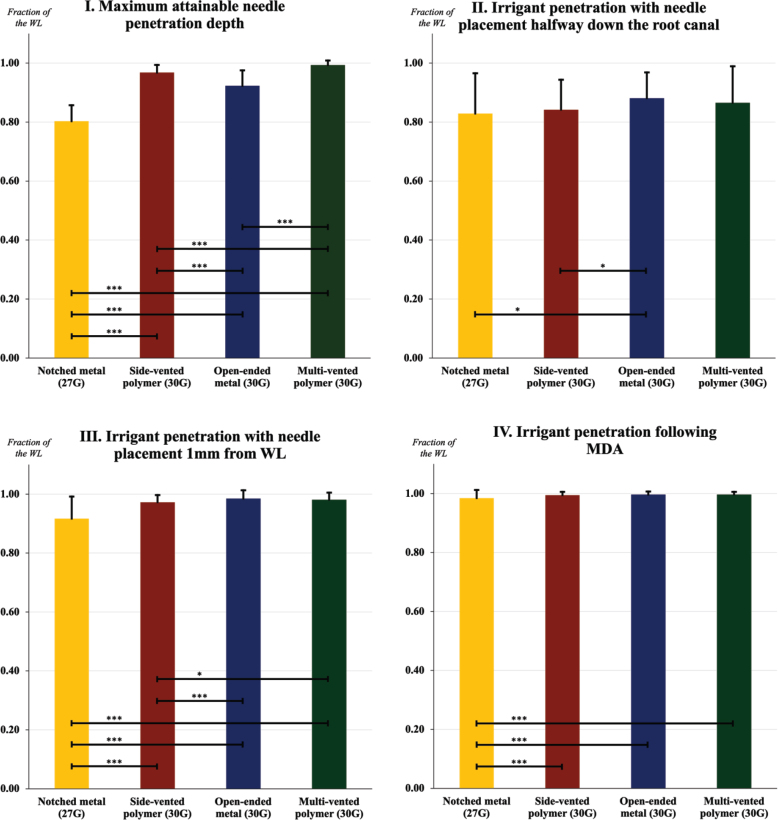
Mean values (expressed in fractions) for each sub-experiment illustrated with standard deviations. Interconnecting lines represent a significant difference between two needle tips. *** *p* < 0.001. ** *p* ≤ 0.01. * *p* ≤ 0.05.

The maximum attainable needle penetration depth ranked from highest to lowest in terms of mean value, was as follows: multi-vented polymer needle (30G), side-vented polymer needle (30G), open-ended metal needle (30G) and notched metal needle (27G). Statistical analysis using Mann–Whitney *U*-test indicated significant differences between all intergroup comparisons (*p* < 0.001) (Figure 5I).

### II. Irrigant penetration with needle placement halfway down the root canal

With needle placement halfway down the root canal, the irrigant penetration was significantly deeper, when using the open-ended metal needle (30G) compared to the notched metal needle (27G) (*p* = 0.042) and the side-vented polymer needle (30G) (*p* = 0.019). However, no significant difference in irrigant penetration was observed between the open-ended metal needle, and the multi-vented polymer needle (*p* = 0.770) (Figure 5II).

### III. Irrigant penetration with needle placement 1 mm from WL

When the needle was positioned 1 mm from the WL, the open-ended metal needle (30G) achieved a significantly deeper irrigant penetration compared to both the notched metal needle (27G) (*p* < 0.001), and the side-vented polymer needle (30G) (*p* < 0.001). Similarly, the multi-vented polymer needle (30G) demonstrated a significantly deeper irrigant penetration than both the notched metal needle (*p* < 0.001), and the side-vented polymer needle (*p* = 0.039). No significant difference in irrigant penetration was observed between the open-ended metal needle and the multi-vented polymer needle (*p* = 0.102) (Figure 5III).

### IV. Irrigant penetration following MDA

Wilcoxon signed-rank test determined that the irrigant penetration was significantly deeper, when syringe irrigation with needle placement 1 mm from the WL was combined with MDA, compared to syringe irrigation alone, regardless of needle tip (*p* < 0.001) (Figure 5IV).

## Discussion

The aim was to compare the needle and irrigant penetration depth using a newly developed needle prototype to three established needle tips, before and after MDA, the null hypothesis being that the irrigant penetration would be the same regardless of needle tip, and the addition of MDA would make no difference in terms of irrigant penetration.

The results of this study showed that the notched metal needle (27G) achieved the lowest needle and irrigant penetration depths. This underlines the impact of needle tip size, in terms of effectiveness of syringe irrigation, as previous studies have indicated that larger needles (21–25G) often fail to reach beyond the middle third of the root canal [17], leading to inadequate disinfection. In this study, needle penetration also varied significantly between the thinner needles (30G). Notably, the multi-vented and side-vented polymer needles (30G) penetrated deeper than the open-ended metal needle (30G). This underlines the statement that a non-metallic needle tip is more beneficial when it comes to curved canals, as the flexibility allows for penetration past severe curvatures, without risking fracture or binding [18]. However, despite the deeper needle penetration with polymer tips, the greatest irrigant penetration was achieved with both the open-ended metal needle (30G) and the multi-vented polymer needle (30G), highlighting the importance of needle design.

The results are consistent with previous studies, emphasizing needle design being a decisive factor in terms of irrigant delivery. Several studies have shown that open-ended needles can achieve deeper irrigant penetration, through higher pressure directed apically, facilitating irrigant delivery to the apical third of the root canal [10, 19]. The open-ended metal needle (30G) in this study exhibited a similar result, achieving a deeper irrigant penetration compared to the side-vented polymer needle (30G), despite a lower needle penetration depth. Consequently, the reportedly extended irrigant flow, is not without an increased risk of apical extrusion [19]. The risk of extrusion was not directly assessed in this study, although it is implied.

On the contrary, the irrigant penetration achieved using the side-vented polymer needle was lower compared to the other designs, which aligns with previous studies that have shown these types of needles to be less effective at reaching the apical thirds of the root canal. The reason for this being the laterally directed flow, which in benefit, also reduces the risk of apical extrusion [20]. The side-vented needles may thus be better suited for final disinfection in straight or slightly curved canals, where the risk of extrusion is higher.

The multi-vented polymer needle performed well in terms of irrigant penetration, without any significant difference compared to the open-ended metal needle. Although both the open-ended metal needle and the multi-vented polymer needle had a significantly deeper irrigant penetration compared to the side-vented polymer needle, the latter one was to a smaller extent, despite being similar in size. This might suggest that the multiple exit vents and/or the trefoil-cross section shape in the multi-vented polymer needle, may lower the irrigant pressure, consequently leading to a decreased irrigant penetration. This indicates that needle tip design has a significant impact on the basic flow developed in the root canal during irrigation [3].

The significant improvement in irrigant penetration shown in this study, when MDA was applied, is consistent with previous studies that have confirmed the efficacy of dynamic activation techniques. MDA can help break down biofilm and dislodge debris, by creating a more turbulent flow, enhancing the overall disinfection of the entire root canal system, especially in areas that typically present a challenge, such as the apical third of the canal. Studies have shown that dynamic activation techniques, albeit MDA or ultrasonic activation, facilitate a more vigorous flow and enhances the irrigant penetration in the apical third [9, 12]. The findings of this study reinforce this notion, as MDA was proven to significantly increase the irrigant penetration, regardless of needle type. However, studies have also shown that all activation methods are associated with the risk of apical extrusion [21]. Irrigant activation methods should therefore be used cautiously, and only in cases of anatomic complexities, where activation methods in general have been proven to be beneficial [17].

## Study’s limitations

While several variables, such as curvature, canal length, connection status, natural morphology and the apical vapor lock effect, were considered, the notable limitations to this study must also be addressed. Sodium hypochlorite is mostly used as an irrigant in a clinical setting [3]. The radiopaque solution used as a surrogate in this study has a higher density and viscosity (2.34 at 37°C), making it an unreliable proxy [8], although the results of this study are more relevant for internal comparisons between the tested needle tips.

Another limitation lies in the use of static, two-dimensional radiographs that are not able to capture changes in pressure, temperature, vibrations and buoyancy, all of which have the ability of influencing the irrigant distribution. The formation and movement of air bubbles within the canal, can also be an altering factor, when determining irrigant penetration through static radiographs [22]. High-speed imaging is an alternative solution to the above-mentioned limitations [8]. Although as this is an optical method, it requires transparent artificial root canals as test subjects. One could argue that this is a great disadvantage, as artificial root canals cannot replicate the complexities of a natural root canal system, making them an unreliable substitute for natural teeth.

Lastly, a direct correlation between irrigant penetration and the healing of apical periodontitis has not yet been confirmed [8]. A recent S3 review highlighted the lack of clear evidence supporting the efficiency of adjunct therapy, such as irrigant activation techniques, light-mediated root canal disinfection (photo-activated disinfection and direct laser irradiation) or ozone therapy, for optimization of root canal cleaning and disinfection [23].

Despite the advancements in irrigation techniques, more research is needed to definitively determine how these factors contribute to the healing process and the prevention of apical periodontitis, which remains the primary success criteria for a root canal treatment [24, 25].

Taken together, there is a need for further research into optimal strategies for endodontic irrigation. A better understanding of the complex interplay between irrigant delivery, canal anatomy, and the healing process is essential for improving root canal treatment outcomes, and thereby ensuring the long-term success of endodontic procedures.

## Conclusion

The findings support the notion that needle design is a decisive factor in terms of irrigant penetration. The multi-vented polymer needle and the open-ended metal needle showed superior performance in terms of irrigant penetration, however the irrigant penetration only managed to reach the working length when MDA was added. The findings of this study reinforce the importance of selecting appropriate needle design and incorporating dynamic activation techniques to optimize the disinfection procedure, particularly in intricate cases.

## Data Availability

Upon request data can be shared.
